# A Multi-Partner Effort to Launch and Sustain Wastewater Surveillance, United States

**DOI:** 10.3201/eid3213.260221

**Published:** 2026-08

**Authors:** Jonathan Yoder, Nicole Fehrenbach, Amanda Raziano, Joseph Kanter, Deise Galan, Rebecca Rainey, Anna Mehrotra, Janet Hamilton, Ewa King, Sarah Wright, Alexandria Boehm, Marlene Wolfe, Rory Welsh, Rachel West, Scott Santibanez, Margaret A. Honein

**Affiliations:** Centers for Disease Control and Prevention, Atlanta, Georgia, USA (J. Yoder, N. Fehrenbach, A. Raziano, R. Welsh, R. West, S. Santibanez, M.A. Honein); Association of State and Territorial Health Officials, Arlington, Virginia, USA (J. Kanter); National Association of County and City Health Officials, Washington, DC, USA (D. Galan, R. Rainey); Water Environment Federation, Alexandria, Virginia, USA (A. Mehrotra); Council of State and Territorial Epidemiologists, Atlanta, Georgia, USA (J. Hamilton); Association of Public Health Laboratories, Bethesda, Maryland, USA (E. King, S. Wright); Stanford University, Stanford, California, USA (A. Boehm); Emory University, Atlanta, Georgia, USA (M. Wolfe)

**Keywords:** Wastewater, viruses, bacteria, parasites, infectious diseases, partnerships, surveillance, United States

## Abstract

The National Wastewater Surveillance System represents a groundbreaking initiative aimed at enhancing public health surveillance for infectious diseases through wastewater analyses. We have outlined current and planned collaborative efforts between the Centers for Disease Control and Prevention and key partners, including academic institutions, utilities, jurisdictions, and public health partner networks, to develop, implement, and sustain wastewater surveillance for infectious diseases to inform public health activities. By addressing critical challenges in assay development, community and clinician engagement, governance structures, public health ethics, data system development and integration, and innovation fostering, those partnerships lay the groundwork for a robust public health surveillance framework. Establishing this novel public health surveillance capability required developing new partnerships and skills, incorporating basic principles of public health surveillance, validating assays, and envisioning a system to meet current and future public health challenges. This capability will foster improved public health responses nationwide.

Public health surveillance is essential for monitoring health trends, detecting outbreaks, and informing and evaluating public health responses. A critical public health innovation from the COVID-19 pandemic was the establishment of wastewater surveillance (WS) for infectious diseases in communities across the United States. WS detects pathogens anonymously in community wastewater to increase understanding of community disease spread and transmission. This system complements traditional public health surveillance that relies on clinical testing of symptomatic patients who seek medical care and syndromic surveillance during patient visits to medical facilities. Because WS is a relatively new capability, the Centers for Disease Control and Prevention (CDC) worked with key partners to address multiple challenges to effectively implement and sustain a national public health WS system; validating laboratory assays that could reliably detect pathogens in wastewater and developing reasonable signal thresholds; establishing a network of wastewater utility partners to provide samples; setting up data systems that provide real-time, bi-directional data; increasing WS capacity and training for the public health workforce; developing the science base, innovation, and ethical framework for WS; and providing response tools for state, territorial, local, and tribal (STLT) health departments to respond to infectious disease threats detected through WS, including tools for engagement with local communities and healthcare providers.

The success of adding WS as an innovative public health tool with applicability far beyond COVID-19 is only possible because of the collaborative efforts of multiple stakeholders. Each stakeholder contributes unique expertise and resources to the continued development of this innovation (Figure).

**Figure:**
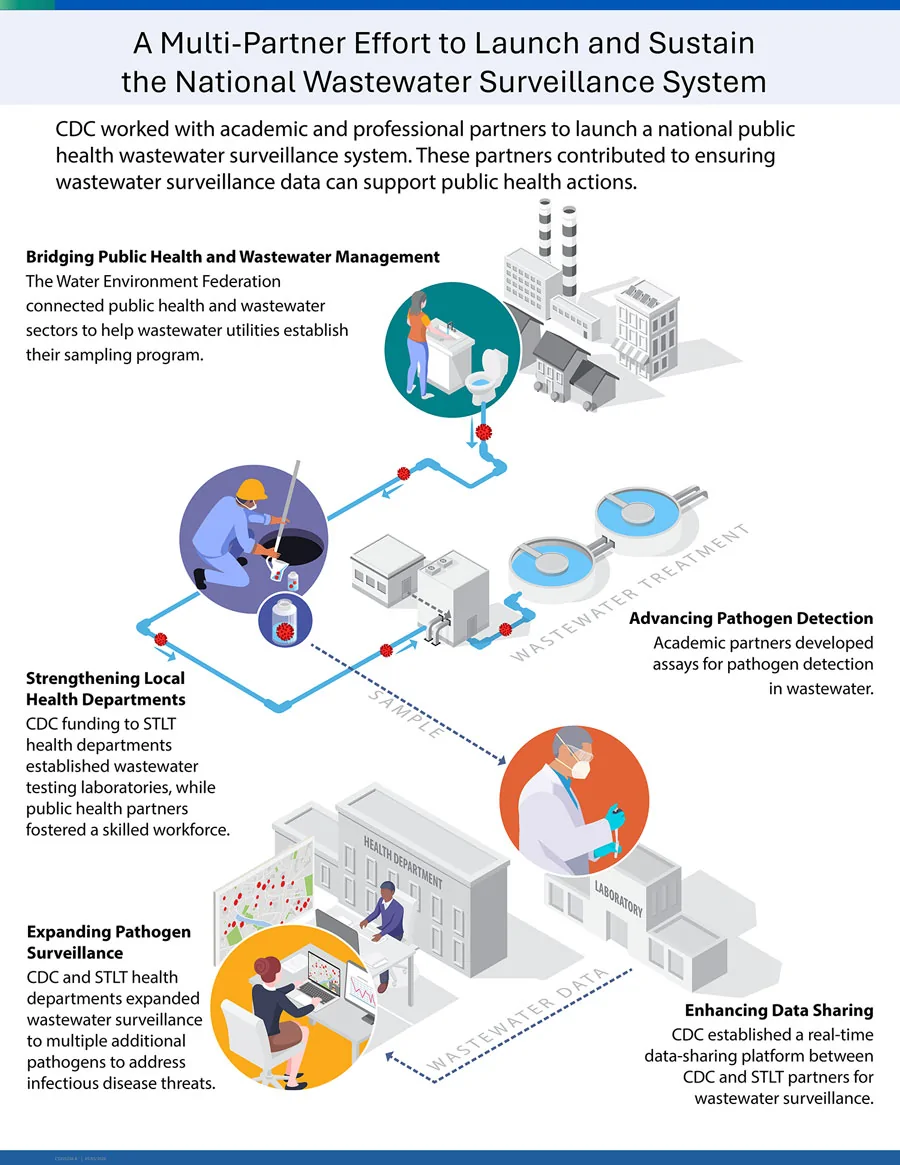
Visualization of the connections between the multiple partners needed to implement and sustain wastewater surveillance to aid public health actions in the United States. CDC, Centers for Disease Control and Prevention; STLT, state, territorial, local, and tribal.

## Partnerships to Address Key Challenges

### Validating WS Assays

Developing specific and sensitive molecular assays for detecting pathogens in wastewater was a substantial challenge. Existing clinical assays needed to be evaluated and adapted for the complex and variable wastewater matrix. In addition, many clinical assays were not in the open knowledge domain, limiting understanding of possible cross-reactions in a complex wastewater matrix. Academic and independent research partners, such as the Research Coordination Network members and WastewaterSCAN, a national program based at Stanford and Emory Universities, played a crucial role in this phase by using their speed and research capabilities to develop, pilot, and validate assays. Concurrently, several state and local public health laboratories began developing analytical capabilities and nurturing relationships with wastewater and public health partners to expand WS nationally. Public health partners field-tested those assays by using their applied technical expertise and resources to ensure the assays were accurate and met the necessary standards for public health surveillance and the public health actions associated with detections. The WS Centers of Excellence, launched in August 2022 and led by public health departments and academic partners located in California, Colorado, Texas, New York, North Carolina, and Wisconsin, became learning laboratories for the development and evaluation of assays for new pathogens. Together, those partners established a framework for continuous assay improvement, incorporating feedback from field applications, transparent sharing of available assays, assay validation, and ensuring a nimble response to current infectious disease threats. This approach proved effective during recent outbreaks of monkeypox, influenza A(H5), and measles. Because the Houston Center of Excellence (Houston, TX, USA) developed an assay for measles, they were able to deploy it promptly in Texas and New Mexico. WastewaterSCAN was able to quickly develop, validate, and deploy an assay nationally. CDC used those assays to rapidly implement national surveillance while generating additional validation data and distributing to national testing partners.

### Building Relationships with Wastewater Utilities

The involvement of wastewater utilities was essential to the success of WS because WS is not feasible without their routine collection of samples. The Water Environment Federation was a pivotal partner in bridging the gap between the public health and wastewater sectors. Through cross-sector workshops, an annual national wastewater disease surveillance summit, training programs, a community of practice, development of a toolkit to inform health departments collaboration with utilities, podcast episodes and a video series, a utility-focused Web site, and a free autosampler and flow meter initiative, the Water Environment Federation enabled introductions across disciplines and lowered barriers to entry for utilities joining WS efforts. The collaboration enhanced the quality of data collected and promoted a shared understanding of the necessity of WS in public health.

### Data Systems for Bidirectional Communication

A robust data infrastructure was necessary to enable bidirectional communication between CDC and STLT health departments, on the basis of the foundational concept that the “basis of effective surveillance is the current and accurate 2-way flow of information among all those who need to know” (*1*). Before the COVID-19 pandemic, public health surveillance platforms were not designed for wastewater data, which includes metadata on the sample, spatial sewershed data, and the ability to collect, analyze, and provision the data in real time to frontline health departments. WS was established on a platform that enabled real-time data sharing between CDC and STLT partners, had the ability to rapidly set up data pipelines for new pathogen targets, and was in alignment with CDC’s Data Modernization Initiative (*2*). National public health partners provided guidance on data standardization and interoperability and convened key state and territorial stakeholders to ensure data were available for public health response. The development of plain-language data dashboards enabled the public and public health jurisdictions to visualize trends, make data meaningful for public audiences, and respond promptly to emerging public health threats. This system exemplified the principles of public health surveillance by promoting timely data sharing and actionable insights and communicating findings for public health action (*3*,*4*).

### Increasing Capacity, Knowledge, and Training for a WS Workforce

A critical component of WS for infectious diseases is the public health capacity to test and analyze data and interpret results in a way that informs public health action. Funding was provided directly to state, territorial, local, and tribal health departments to establish their wastewater testing laboratories and surveillance programs. Public health partners provided input to guide capacity building by mentoring, developing standards, and addressing public health ethics (*5*). Academic and clinical partners, including those associated with the WS Centers of Excellence, equipped public health professionals with the necessary skills in data interpretation, laboratory techniques, and community and clinician engagement strategies. Communities of practice developed by partner organizations were instrumental in helping the new utility, laboratory and health department workforce connect and share successes and challenges. The Association of Public Health Laboratories (APHL) Wastewater Surveillance Laboratory Community of Practice started in 2020 as a small group of laboratories who met monthly to discuss the challenges of onboarding this testing; it has grown to include 643 members. Monthly presentation- and discussion-based calls cover all aspects of laboratory implementation of WS, including new targets for testing, workflow efficiencies, and sequencing. Other workforce development efforts include APHL and Council of State and Territorial Epidemiologists peer-to-peer network programs that provided bidirectional in-person visits between jurisdictions to learn new techniques and establish collaborative relationships. Those development efforts include APHL fellowship and internship programs that have sponsored 432 fellows and 298 interns in state and local public health laboratories.

Throughout the United States, local and state health departments are working in collaboration with utilities, academic institutions, and private partners to advance WS. Local health departments (LHDs) play a critical role in those efforts by using wastewater data to inform public health action. Although some LHDs lead WS activities and manage testing directly, others play a more supportive or coordination-based role; however, all contribute critically to developing a comprehensive WS system.

To strengthen local capacity, the National Association of County and City Health Officials (NACCHO), with support from CDC, launched the Wastewater Surveillance Mentorship Program. NACCHO identifies LHDs that have extensive WS experience (mentors) and pairs them with LHDs in the early stages of program development (mentees). Throughout the program, mentors provide tailored technical assistance to help mentees design, implement, and strengthen their WS programs on the basis of their specific goals and jurisdictional needs.

To further expand support and create wider access to WS expertise, NACCHO launched the Fostering Local Wastewater Monitoring Network, a community of practice designed for LHD staff at all experience levels. By investing in workforce capacity in frontline public health, health departments can effectively use wastewater data to inform public health decisions and align with the principles of public health surveillance that continually adapt to evolving health challenges (*6*,*7*).

### Developing the Science Base, Innovation, and Ethical Framework

To meet emerging and reemerging infectious disease threats, WS requires continual innovation. Although it began as an innovative system to provide critical data on community levels of COVID-19, WS has expanded to numerous pathogens on the basis of the public health need, most recently including influenza, monkeypox, and measles virus. Innovation requires continued engagement with academic partners such as WastewaterSCAN and the Centers of Excellence to expand the science base.

The collective expertise of academic partners, public health organizations, and wastewater utilities fosters an environment that is conducive to experimentation and adaptation but also promotes ethical public health practice. The Association of State and Territorial Health Officials engaged with CDC and the Centers of Excellence to develop an applied ethical framework (*5*). This framework offers guidance to utility partners, health departments, academic partners, and policymakers involved in protecting privacy, preventing stigma, ensuring proper data stewardship, and helping build trust with contributing communities and clinical professionals to enable the effective use of wastewater data for infectious disease surveillance (*8*,*9*). A strong focus on partnerships enabled the US WS program to embrace new technologies and methodologies and demonstrate leadership in public health innovation, which is a key marker of successful public health innovation (*6*,*7*).

### Supporting Public Health Action

The core purpose of public health surveillance is effective and timely public health action. Public health partners provided key support to ensure that wastewater signals informed public health action for each new pathogen target. They developed response checklists, public communication strategies, and data visualizations that enabled health departments to use wastewater data for the early detection of outbreaks, monitoring persistent infectious disease threats, assessing public health interventions, and providing data that the public can use to inform and protect themselves. Partners provided ongoing feedback and input to develop and continually refine those public health action tools.

## Future Challenges

The launch of the National Wastewater Surveillance System in the United States underscores the importance of collaborative partnerships in addressing complex public health challenges. By leveraging the respective infrastructure and strengths of diverse partners and working to build community trust, WS enhances our ability to monitor and respond to infectious disease public health threats. Continued innovation in testing methods (e.g., sequence-based or metagenomic approaches), data analysis, and use of wastewater for public health action will be essential to adapt to evolving public health needs and ensure the effectiveness of surveillance efforts. Integration of wastewater data into communication with key partners such as clinical professionals will extend the benefit of these data to protect the health of their patients.

Data sharing among different parties is critical to advance the use of data for public health action and advance science; data sharing protocols are needed to ensure data are used responsibly and to benefit society. Whereas clinical laboratories generally use US Food and Drug Association–approved methods for diagnostics, WS requires new approaches to ensure data quality. Wastewater matrices are extremely complex and diverse; sometimes specific samples require bespoke methods for analysis. Because methods might be more complicated, it is essential for groups to make methods publicly available to aid data interpretation and advance wastewater-based epidemiology science. Biobanking of wastewater samples is practiced by some groups but not others. Biobanked samples are extremely valuable for retrospective studies, including studies that test new assays, but there are ethical and legal issues associated with the practice that should be addressed. Some sequence-based and metagenomic approaches are being applied to wastewater and might greatly expand the scope and value of WS; those approaches need refinement as public health surveillance tools (*10*).

In conclusion, WS is an emerging field that requires continued investment and robust partnerships to reach its full potential. WS aligns with the vision of modern public health surveillance that is adaptable, integrated, and capable of addressing emerging health threats (*6**,**11*,*12*). Integration of WS into existing pathogen and genomic public health surveillance systems is an opportunity to fundamentally transform our understanding of infectious disease transmission within communities. 
